# Engagement in Evidence-Based Practice Activities and Thinking by Rehabilitation Therapists: Predictive Factors

**DOI:** 10.1016/j.arrct.2025.100542

**Published:** 2025-10-28

**Authors:** Angela Benfield, Mark V. Johnston, Cheryl Miller, Alexandra E. Harper, Minmei Shih, Elizabeth R. Skidmore

**Affiliations:** a University of Wisconsin-La Crosse, La Crosse, WI; b University of Wisconsin-Milwaukee, Milwaukee, WI; c Therapy Operation, Encompass Health Corporation, Florida; d Institute for Clinical & Health Research, University of Michigan, Ann Arbor, MI; e University of Pittsburgh School of Health and Rehabilitation Sciences, Pittsburgh, PA

**Keywords:** Clinical competence, Clinical reasoning, Evidence-based practice, Health occupations, Implementation science, Rehabilitation

## Abstract

**Objective:**

To explore relationships between therapists’ recurrent, self-regulated evidence-related activities and antecedent factors that may influence these habits.

**Design:**

Secondary analysis of survey data using multivariate regression/correlations analyses.

**Setting:**

Representative sample of 30 inpatient rehabilitation clinics across the United States.

**Participants:**

163 rehabilitation therapists (N=163), specifically physical and occupational therapists and speech-language pathologists.

**Interventions:**

Not applicable.

**Main Outcome Measures:**

Evidence-informed professional thinking, which is comprised of measures of recurrent, self-regulated evidence-informed practice (EIP) and reflective critical clinical reasoning (CCR) activities by therapists.

**Results:**

Scores indicate infrequent engagement in EIP activities (mean=4.22, SD=0.93), with somewhat greater engagement in CCR (mean=5.00, SD=0.920). Therapists generally had positive attitudes toward evidence-based practice (EBP) on the Evidence-Based Practice Attitude Scale-36. The EIP and CCR were highly related (*r*=.651, *P*=.001). Even after linear controls for other possible predictors, beliefs that EBPs lead to greater Job Security predicted greater engagement in both EIP and CCR (*P*<.001). Openness was also independently related to engagement in EIP (*P*<.001) and to CCR (*P*<.011), apparently mediated by Feedback, Professional Education, and awareness of Limitations (of EBP). Specialty certification was clearly related to EIP (*P*<.016), but many common educational and experience background variables had weak or nonsignificant relationships with therapists’ EIP or CCR habits.

**Conclusions:**

Self-regulated engagement in EIP and CCR are predicted by identifiable but somewhat complex antecedents. CCR was more strongly related to EIP than any other variable. Results suggest that implementation strategies that engage therapists’ reflective critical clinical reasoning will be more successful at increasing therapists’ engagement in self-regulated EBP habits than in strategies that do not.

Rehabilitation practitioners need to integrate current best evidence from relevant, controlled clinical research with their clinical experience and patient preferences in making clinical decisions, ie, to employ evidence-based practices (EBP) when possible.^1^, ^2^, ^3^, ^4^ The EBP requires appraisal of the relevance and strength of research evidence and also requires clinical expertise and reasoning skills to apply evidence to treat individual patients or clients.^4^, ^5^, ^6^ While health professionals are increasingly mandated to engage in EBP, studies have reported that EBP of rehabilitation therapists remain inconsistent and limited.^7^, ^8^, ^9^, ^10^ In addition, few studies have included measures of both EBP itself and related clinical reasoning habits among rehabilitation therapists. Consequently, knowledge of factors influencing the recurrent engagement of rehabilitative therapists in EBP is limited.^11^, ^12^, ^13^, ^14^

Research has examined attitudes about EBP, workplace climate, and practitioner characteristics (ie, years of experience, educational attainment, or specialty certification) as factors possibly related to evidence uptake.^12^^,^^15^^,^^16^ These studies have provided valuable information and have identified factors that support uptake of specific EBPs.^17^ The EBP uptake, however, remains limited. Therapists resist some approaches, and frequency and fidelity of use over time often declines.^6^^,^^11^^,^^14^^,^^18^ While such studies have provided valuable information,^17^ a characteristic of most of these studies has been a focus on uptake of specific pre-established EBPs and use of a top-down educational or learning strategy. New approaches to enhancing evidence application in clinical practice are needed.

Few quantitative studies of therapists’ professional habits of engagement in evidence application and on related clinical reasoning have been performed; empirical knowledge of antecedents associated with such desirable activities remains very limited.^19^, ^20^, ^21^ Greater understanding is needed of best approaches and strategies to increase professional engagement in evidence-informed practice (EIP) and good critical clinical reasoning (CCR).

The EBP requires more than appraisal and knowledge of best evidence: it requires clinical expertise to apply the evidence to individuals in clinical practice. Furthermore, a “conscientious, explicit, and judicious” reasoning process is required.^1^^,^^22^ Clinical expertise and reasoning skills are necessary to choose, apply, and adapt relevant evidence to individuals in actual clinical practice. Developing good clinical reasoning and expertise, however, is not always a given; recurrent or habitual engagement in reflective reasoning about evidence application and clinical experience is needed.^5^^,^^23^, ^24^, ^25^^,^^26^ Professional clinical reasoning has also been described as the cognitive process involving reflective, abductive reasoning used to diagnose and manage a clients’ health problems. Such reflective or explicit cognitive processes also enhance learning from clinical experience.^5^^,^^26^

Improving and sustaining the quality and effectiveness of care and treatment provided by rehabilitation therapists requires their engagement in both EBP-related activities and clinical reasoning as well as learning by both individuals and organizations.^1^^,^^2^^,^^27^ This study addresses self-regulated evidence-related activities used to highlight the self-initiated activities of professionals to identify relevant evidence and apply it to their clients/patients, to evaluate the outcomes, and to reflect on practice to improve future practices. Therapists need to reflect on both written guidelines and their own abductive, deductive, and inductive clinical reasoning and performances.^28^

Many studies focus on uptake of specific EBPs and implementation fidelity or on therapists’ technical knowledge and personal or technical skills.^29^^,^^30^ Such studies are valuable and needed. The total process of provision of high quality effective care, however, involves more than adherence to guidelines and manualized protocols.^1^^,^^6^^,^^22^ Clinical reasoning and skills are necessary to adapt and modify evidence to the wide variety of diagnoses, comorbidities, functional strengths and weaknesses, preferences, and circumstances of individuals served.^1^, ^2^, ^3^, ^4^^,^^26^

More generally, clinical practice involves abductive reasoning. Abductive reasoning involves seeking the most plausible or likely explanation for observations. It involves an interplay between inductive reasoning from observation, formulating relevant alternative theories or explanations, deduction from these explanations, often leading to further observations or testing, leading to choice of best explanation and treatment strategy.^31^^,^^32^ The processes involves forming educated guesses or theories about from available information about the patient/client, formulating hypotheses, and making further observations (for instance, about client/patient diagnoses, symptoms, strengths, and weaknesses) to check which explanation is most plausible or supported. A treatment strategy is attempted, and client responses (beneficial and not) are observed or measured, which may lead to further changes in treatment strategy and client observation.

Quantitative research on self-regulated practices addressing both evidence-related and clinical reasoning practices by rehabilitation therapists is quite limited. Most of the literature on the topic is qualitative, descriptive, or theoretical.^19^^,^^29^^,^^33^^,^^34^ A major reason why quantitative research on the topic has been rare is that, until recently, there has been no valid and reliable measure of engagement in the occupational habits involved in both processes of evidence-related and clinical skill-related reasoning processes.

A measure of evidence-informed professional thinking (EIPT) has rather recently been developed that addresses rehabilitation therapists’ engagement in both application of external evidence and related professional clinical reasoning from clinical experience.^21^ The EIPT tool is comprised of 2 related subdimensions—EIP and CCR.^21^ The EIP measures frequency and engagement of therapists’ reported recurrent activities or habits related to learning and applying external evidence to their clinical practices. The word “informed” was used because the model of EIPT is a systems-based model that considers professional role/responsibilities, research awareness, decision-making, and critical thinking, which inform the development of client-specific interventions. In addition, basing all rehabilitative therapies only on strong evidence is not realistic, given the limited number of relevant randomized clinical trials (RCTs).^21^ Note that EIP and CCR focus on therapists’ habitual activities, not on the implementation of any set of specific EBPs, although many educational programs, policies, and studies require such a specific focus.

The CCR queries recurrent activities or habits related to therapists’ reflecting on their own clinical experiences and choices. Such explicit or critical reflection is needed in health care. One estimate is that 50% of clinical behaviors exhibited by health professionals are done via nonreflective processes.^24^ Recurrently basing clinical decisions on pragmatic tradition, habits of obedience, intuition, or whatever appeals leads to lower EIP and CCR scores. The routine professional reasoning processes of clinicians are important both for application of best evidence and learning from experience.

While EIPT itself has been previously studied,^7^^,^^21^^,^^35^ its relationships to important antecedent factors (eg, therapy discipline, years of experience, facility size, organizational climate, or therapists’ attitudes) have not previously been studied. The present study addresses this lacuna.

Broadly, the present study attempts to provide knowledge to support new approaches to study and improve the quality of rehabilitative care by focusing on therapists’ self-regulated engagement in evidence application activities and related reflective clinical reasoning processes. More specific aims are to:

1. Test whether results of previous studies of EIPT generalize. Specifically, does the high correlation between CCR and EIP found previously (*r*=.78, *P*=.001)^21^ generalize to a new sample of rehabilitation therapists?
2. Identify background factors, clinic characteristics, and therapist characteristics and attitudes that are reliably and independently related to engagement in self-regulated EIP and CCR. We hypothesized that EIP would be significantly related to ≥2 subscales of the Evidence-Based Practice Attitudes Scale (EBPAS-36), namely, openness (to trying new, research based, or manualized interventions) and job security (beliefs that EBP activities will help to maintain and enhance employment).
3. Develop greater understanding of factors or processes related to therapists’ engagement in self-regulated EIP and CCR to guide future research and practice on methods of improving use of relevant evidence in practice and thus improving clinical competence and care quality and effectiveness.

## Methods

This study is a secondary analysis of data collected for larger study on readiness for the implementation of EBP called strategy training (NIH UL1TR001857). Ethical approval was granted by Pittsburgh IRB STUDY2111p0p71. All procedures were approved by the University of Pittsburgh and University of Texas Institutional Review Boards. The participants consented to participate in the study. Therapists and therapy directors within 30 inpatient rehabilitation facilities were contacted for participation from 147 facilities within a large national for-profit rehabilitation corporation. Facilities were selected to ensure heterogeneity with respect to facility location (national representation), size, and patient population (race, socioeconomic status, insurance provider). Written informed consent was obtained from all 30 directors and 173 (of 180) therapists approached. Two occupational therapy, 2 physical therapy, and 2 speech-language pathology therapists were approached at each facility. For this study, only data from direct care therapists (as distinguished from directors) were analyzed. Therapists completed an anonymous internet survey that asked about therapist and facility characteristics, EIPT (ie, EIP, CCR), and the EBPAS-36^36^ using REDCap^a^.

### Instruments

The primary outcome measure used was the EIPT, which is comprised of 2 separate but related measures of professional practice: EIP and CCR.^21^ The EIP consists of 16 items probing frequency and engagement in clinical activities necessary for EBP.^21^ The EIP activities measure has a person separation reliability (similar to a Cronbach’s α) of 0.90 and is able to reliably distinguish 4 different 90% levels (strata) of performance, ranging from “in need of support,” “basic competency,” “competent,” to “high competency.”^21^

The CCR measures recurrent activities or habits of engagement involved in learning from clinical experience, including the experience of other clinicians. The CCR consists of 16 items on therapist habits of engagement in abductive, professional clinical reasoning.^21^ The CCR measure has a person separation reliability of 0.90 and can reliably distinguish 4 different 90% strata, ranging from “in need of support” (mean scores <3.5) to “high competency” (mean scores >5.5).^21^ CCR and EIP are related conceptually and empirically.^21^

As therapists go through a reflective, abductive reasoning process, explanations or theories about a client’s problem should improve more than if they rely on nonreflective processes such as intuition, following instructions, or tradition.^24^^,^^28^ The EIP and CCR emphasize basic reflective processes but do not assess more advanced, specialized reasoning or knowledge processes valuable for specific client problems.

Both EIP and CCR subscales of EIPT generally use a 5-point rating scale of frequency for items (raw scores): 1 = Never; 2 = Rarely (1-4×/mo); 3 = Sometimes (5-8×/mo); 4 = Frequently (9-12×/mo); 5 = Almost always (daily). Using standard Rasch analysis procedures,^21^^,^^37^ raw scores are rescaled so they can be logically added to construct maximally accurate (but still probabilistic) equal-interval measures of items relevant to the content domain. Scores on the 2 summative measures—EIP and CCR—were rescaled to range from 0-10, with higher sum scores indicating greater engagement in the activities.^21^ The approach behind the EIPT differs from that usually employed in implementation research. The conceptual, occupational framework and research behind the EIPT may be unfamiliar to readers. Hence, we provide additional information below. For even more complete information, please read Benfield and Johnston (2020).^21^

EIPT items were developed from prior literature (eg, Higgs^3^; Melnyk and Fineout‐Overholt^38^) and testing with rehabilitation providers (nursing, clinical social workers, occupational therapists, physical therapists, and speech-language pathology professionals). The EIPT is a self-report measure, and different and more precise measures, potentially using observational checklists, would be needed to measure delivery of specific needed EBPs. At the same time, the total extent of general evidence-related activities or engagement of therapists needs empirical assessment. The recurrent activities of therapists to *engage* in EBPs and apply them to individuals treated, however, are approximately but demonstrably measurable using the EIPT.^21^ At 32 items in length, the EIPT is also practical to use to orient interventions and therapists to evidence-related and clinical reasoning issues.

Within the domain of items developed, Rasch analysis was used to identify items that probabilistically measured a common thread of recurrent, self-regulated activities or habits related to identifying and reflecting on external evidence (EIP) and on learning clinical practice and adapting therapy to individuals served (CCR) by rehabilitation therapists. The psychometric structure of EIPT been replicated across different samples of rehabilitation therapists, including therapists in countries outside the United States.^7^^,^^21^

Therapists’ attitudes toward EBP generally and related organizational climate factors were assessed using the EBPAS-36.^36^ The EBPAS-36 consists of 36 items. Agreement with each item is rated using a 5-point Likert-type scale of agreement scored as follows: “not at all” (1), “slight extent” (2), “moderate extent” (3), “great extent” (4), or “very great extent” (5). Higher scores indicate more positive attitudes toward EBP.^36^ The EBPAS-36 has 12 subscales of 3 items each probing attitudes and opinions that theoretically facilitate or hinder the implementation of EBPs. Several subscales expressing disagreement with EBP (Divergences, Limitations, Monitoring, Balance, and Burden) require reversing the sign to estimate overall attitudes. The EBPAS-36 has moderate internal consistency (Cronbach α=0.79); subscale reliability varies between 0.65 for Divergence and Balance and 0.89 for Requirements.^36^ The EBPAS-36 has been widely used, but construct validity studies on it have reported somewhat mixed results.^39^

Therapist characteristics queried included sex, discipline (occupational, physical, or speech and language therapy), education level and type, number of specialty certifications, and years of experience. Facility size (beds) was also recorded.

### Statistical analysis

Statistical analysis proceeded from simpler descriptive statistics to bivariate and then to multivariate analyses using SPSS.^b^ To organize analyses of a complex set of variables, it is important to group variables in some meaningful way. Grouping by temporal occurrence enables results to provide interpretable clues or hypotheses to guide future research on the processes leading to subsequent outcomes. First, we examined the relationship between CCR and EIP. Bivariate correlations between these 2 variables and therapists’ perceptions of EBP-related workplace attitudes were examined next (EBPAS-36), followed by more temporally distal factors (eg, therapist education, years of experience, licensure, certifications) and facility size. We then used exploratory multiple regression/correlation to provide an overview by identifying the strongest factors independently predicting both EIPT dimensions, considering collinearities.^40^ Forward stepwise selection and backward elimination procedures were both used to analyze predictive correlations to identify the most reliable relationships. Stepwise forward regression tests possible predictors, analyzing the strongest first, semipartialling out linear effects on other possible predictors, and then analyzing the next strongest predictor. While forward stepwise correlations may seem simpler, backward elimination provides a more conservative and sometimes more insightful result, as it controls for relationships with a larger set of collinear predictors; it begins by entering all possible predictors into the equation and then eliminating those that do not have a significant effect on prediction of the dependent variable. Default entry and elimination criteria in SPSS were used.

## Results

### Sample description

Of the 180 therapists approached, 173 provided informed consent; of these, 163 completed the survey. As shown in table 1, the sample was comprised of 62 (38%) occupational therapy practitioners, 56 (34.7%) physical therapy practitioners, and 43 (26.4%) speech-language pathologists. Mean years of total rehabilitation experience were 9.5 years (range, 0.8-28y) and 6.3 years for rehabilitation hospital experience (range, 0.75-28y) for participating therapists. The great majority (95%) of respondents had 1 or 2 specialty certifications, with a few (3.1%) having 3 or more. Sample size is 161-163 in further analyses unless otherwise noted.

**Table 1:** Therapist and facility characteristics.

Characteristic	No. (%) of Respondents*
Profession	
Occupational therapist	53 (32.5%)
Occupational therapy assistant	9 (5.5%)
Physical therapist	48 (29.8%)
Physical therapy assistant	8 (4.9%)
Speech-language pathologist	43 (26.4%)
Education	
Associate degree	10 (6.1%)
Bachelor’s degree	16 (9.8%)
Master’s degree	86 (52.8%)
Clinical doctorate	45 (27.6%)
PhD, EdD, etc.	4 (2.5%)
Facility size	
Small (<35 beds)	38 (23.3%)
Medium (35-58 beds)	74 (45.4%)
Large (>58 beds)	51 (31.3%)
Experience (total)	
0-2.9 y	44 (27.0%)
3-5.9 y	35 (21.5%)
6-8.9 y	28 (17.1%)
9-11.9 y	13 (8.0%)
>12 y	43 (26.3%)
Experience in inpatient rehabilitation	
0-2.9 y	59 (36.1%)
3-5.9 y	38 (23.3%)
6-8.9 y	24 (14.7%)
9-11.9 y	11 (6.7%)
>12 y	29 (18.0%)
Specialty certificates	
None	2 (1.2%)
1-2 certificate(s)	156 (95.7%)
3+ certificates	5 (3.1%)
Sex	
Female	148 (85.1%)
Male	24 (14.0%)
Decline to identify	2 (1.1%)

⁎ 161-163 completed items.

### Therapists’ engagement in EIP and CCR

Engagement of therapists in EIP and CCR is described in table 2, both in terms of raw score item ratings (middle column) and in terms of the Rasch-analysis-recalibrated 10-point summative measures of engagement in EIP and CCR (last column to the right). Overall, engagement in CCR was somewhat greater than engagement in EIP: 4.97 vs 4.22. The interpretation of the overall engagement with CCR is “basic competency” because of the infrequent engagement. The interpretation for the frequency of engagement in overall EIP is also basic competency for the same reason (infrequent engagement is indicated by scores between 3.5 and 5.5.^21^

**Table 2:** Mean engagement in EIP and CCR and examples of content items.

Measure and Items	Mean (SD) of Item Ratings of Activity Frequency*	Mean (SD) on Summative Measure†
Evidence-informed practice (EIP)	NA	4.22 (0.90)
Reflected on outcomes data	2.91 (1.19)	3.86 (1.58)
Assessed performance after a specific time	2.50 (1.05)	4.42 (0.46)
Developed a PICO question	2.25 (1.20)	4.96 (1.73)
Search a database or peer-reviewed journal	2.18 (0.92)	3.96 (1.27)
Shared results of outcome measurement	2.17 (1.09)	3.33 (1.38)
Reflected on an article to integrate with experience	2.12 (0.84)	3.64 (1.15)
Sought systematic reviews or meta-analysis	1.83 (0.86)	4.02 (1.20)
CCR	NA	4.97 (0.92)
Shared a clinical experience to gain a deeper understanding (8)	3.61 (0.92)	5.26 (1.51)
Appraised their knowledge	3.41 (0.90)	4.99 (1.57)
Questioned the accuracy of their knowledge and beliefs	3.23 (1.05)	4.49 (1.70)
Developed a clearly answerable question that defines population, intervention, and outcome (PIO)	3.24 (1.11)	5.42 (1.76)
Integrate best practice evidence into their local setting	2.63 (1.03)	4.76 (1.67)
Formally shared evidence with at least 2 colleagues	1.75 (0.89)	4.41 (1.57)

Abbreviations: CCR, critical clinical reasoning; EIP, evidence-informed practice; NA, not applicable; PICO, population, intervention, comparison, and outcome; PIO, population, intervention, and outcome.

⁎ Means of items on 1-5 rating scale of frequency (raw scores).

† Mean of calibrated summative measure, 0-10 point scale.

Considering individual item means, frequency ratings near 2 indicate activities that are “rarely (1-4×/mo)” done; ratings near 3 are done “sometimes (5-8×/mo).” The most frequently performed item in EIP engagement was reflecting on outcomes data (2.91, “sometimes”). Therapists tended to “rarely” search a database or peer-reviewed journal (2.18), share the results of outcome measurement (2.17), or reflect on an article to integrate it with experience (2.12); the least frequently performed EIP item was seeking systematic reviews or meta-analyses (1.83). Among CCR items, the most frequent activity was sharing a clinical experience to gain a deeper understanding (3.61, between “sometimes” and “frequently (9-12×/month)”). Also, they “sometimes” appraised their own knowledge (3.41). Formally sharing evidence or information with a group of colleagues was least frequent (1.75, less than “rarely”).

### Therapists’ attitudes related to EBP and their organization

Table 3 displays average (mean) therapist attitudes in subscales of the EBPAS-36.^36^ Therapists had a generally positive orientation to EBP overall as shown by the overall average ratings 3.93 (a simple average of agreement on items expressing positive views of EBP and inverse scoring of the few subscales critical of EBP). On the EBPAS-36 items, a “4” indicates a “great” extent of agreement. Agreement with most items and subscales was “great.” There was “very great” agreement that therapy practices need to fit individuals treated and fit with therapists’ own philosophy or approach. Lowest agreement was with the Balance subscale (about therapy being a balance of art and science).

**Table 3:** Therapists’ EBPAS-36 attitudes.

Scale or Subscale	Mean ± SD	Agreement Label*	Range
***EBPAS-36 Total Score*** (average of 12 subscales)	3.93±0.36	Great	2-5
**Requirements** (from supervisor, agency, or the state)	4.06±0.95	Great	1-5
**Appeal** (makes sense, colleagues happy with it, enough training*)*	4.38±0.49	Great	3-6
***Openness*** (to therapies that are manualized, developed by researchers, or new)	4.14±0.62	Great	2-5
**Divergence**† (not useful, not use, clinical experience more important)	4.06±0.60	Great	2-5
***Limitations***† (to specific clients with multiple problems, individualization, clinical experience more important)	4.35±0.67	Great	2-5
**Fit** (with clients, own views, own clinical philosophy)	4.59±0.47	Very Great	3-5
**Monitoring**† (work without oversight, etc.)	3.41±0.93	Moderate	1-5
**Balance**† (between art and science, overall clinical competence)	2.74±0.74	Moderate	1-5
**Burden**† (time to learn new things, obligations, fit in EBP)	4.22±0.746	Great	1-5
***Job security*** (incentives to keep job, get new job, find work)	3.03±0.99	Moderate	1-5
***Organizational support*** (continuing education, training, ongoing support)	4.19±0.72	Great	1-5
***Feedback*** (on performance, and supervision)	3.98±0.78	Great	2-5

Abbreviations: CCR, critical clinical reasoning; EBPAS-36 Evidence-Based Practice Attitude Scale-36; EIP, evidence-informed practice.

⁎ Label for the integer nearest to the mean reported.

† Negatively phrased and scored subscales. Subscales correlated with EIP or CCR (compare with Table 4) are italicized.

### Bivariate predictors of EIP and CCR

EIP and CCR were moderately strongly related (*r*=.651, *P*=.002), confirming results of previous studies.^7^^,^^21^ Table 4 describes other simple bivariate correlations between EBPAS-36 subscales and both EIPT measures. EBPAS-36 total score was statistically (*P*<.02) but not strongly correlated with therapists’ engagement in self-regulated EIP and CCR. Five EBPAS-36 attitude subscales were also significantly (*P*<.01) correlated with both EIP and CCR: Openness, Limitations, Job Security, Organizational Support, and Feedback.

**Table 4:** Bivariate correlations of the EBPAS-36 and subscales with EIP and CCR.

	EIP		CCR	
EBPAS-36 Components	ρ (rho)	Pearson *r*	ρ (rho)	Pearson *r*
EBPAS-36 total	.20*	**.27**†	**.26**†	**.33**†
Requirements	.04	.08	−.03	.01
Appeal	−.06	−.02	−.00	.038
Openness	**.25**†	**.27**†	.19*	**.24**†
Divergence	.07	.12	.04	.07
Limitations	.06	.08	.**21**†	**.21**†
Fit	.08	.09	.06	.08
Monitor	−.04	−.01	.07	.08
Balance	.09	.09	.06	.05*
Burden	.12	.17*	.09	.14
Job Security	**.26**†	**.31**†	**.35**†	**.39**†
Organizational Support	.17*	**.21**†	**.25**†	**.30**†
Feedback	.10	.16*	**.24**†	**.29**†

Abbreviations: CCR, critical clinical reasoning; EBPAS-36 Evidence-Based Practice Attitude Scale-36; EIP, evidence-informed practice.

⁎ *P*<.05 (2-tailed).

† *P*<.01 (2-tailed).

Table 5 displays therapist and facility characteristics potentially related to EIP and CCR. Most such variables did not have reliable (statistically significant) correlations with EIP or CCR. The only education factor clearly associated (*P*<.005) with EIP was attainment of specialty certification. Plots showed that the mean engagement in EIPT activities were slightly greater among therapists with bachelor’s and master’s degrees and lower among those with clinical doctorates (eg, OTD, DPT).

**Table 5:** Relationships of facility and therapist characteristics with the EIP and CCR.

	EIP		CCR	
	Statistic	*P*	Statistic	*P*
Facility size (no. of beds)	ρ=.045	.567	ρ=0.39	.624
Sex	t=.281	.154	t=−0.390	.631
Profession (OT, PT, SLP)	F=.171	.953	F=1.234	.299
Education level	ρ=−.077	.329	ρ=−.141	.073
Years of professional experience	*r*=.117	.141	*r=*.068	.388
Specialty certifications	ρ=.217	.005	ρ=.131	.096

Abbreviations: CCR, critical clinical reasoning; EIP, evidence-informed practice; OT, occupational therapist; PT, physical therapist; SLP, speech-language pathologist.

Table 6 presents multiple regression analyses to identify outstanding additive EBPAS-36 predictors, contrasting forward selection procedures to backward elimination. Job Security and Openness were significant (*P*<.01) predictors of EIP. Backward elimination revealed 2 other possible independent predictors of EIP not identified in the forward analysis: first, the belief that therapy is a Balance between art and science; second, a weak inverse relationship of Monitoring indicating that therapists who practice more EIP express greater disagreement to having have someone provide detailed oversight on their clinical practices.

**Table 6:** Multiple regression of the EBPAS-36 subscales on EIP and CCR.

Significant Stepwise Predictors	*R*^2^ at Step (Adjusted *R*^2^)	Standard β	T	*P*
Stepwise forward on EIP				
Job Security	.10 (.09)	.27	3.53	<.001
Openness	.14 (.13)	.22	2.94	<.004
Backward elimination on EIP				
Job Security	NA	.27	3.62	<.001
Openness	NA	.26	3.41	<.001
Balance	NA	.17	2.11	.036
Monitoring	NA	−.15	−1.81	.072
Model (using all 4)	1.7 (1.51)	NA	NA	<.001
Stepwise forward on CCR				
Job Security	0.15 (.15)	.40	4.99	<.001
Openness	0.18 (.17)	.19	2.56	<.011
Backward elimination on CCR				
Job Security	NA	.31	4.03	<.001
Feedback	NA	.16	2.03	.044
Limitations	NA	.14	1.97	.050
Model using all 3	1.96 (1.81)	NA	NA	<.001

Abbreviations: CCR, critical clinical reasoning; EBPAS-36 Evidence-Based Practice Attitude Scale-36; EIP, evidence-informed practice; NA, not applicable.

The lower half of table 6 shows that Job Security was a stable predictor of CCR regardless of whether forward or backward procedures were employed. Openness was a statistically significant (but rather weak) predictor using forward procedures but was removed with backward elimination; positive attitudes toward receiving Feedback on clinical practices and awareness of Limitations of evidence supplanted, making a slightly more predictive model (*R*^2^=.196 rather than .18),

### Analysis using all predictors

Table 7 adds more distal factors—therapist characteristics and facility size—and presents the most embracing summary of independent predictors of EIP and CCR. Both stepwise forward and backward elimination identified Job Security, Openness, and Specialty Certifications as significant independent predictors of EIP. Therapists who more strongly base their decisions on general impressions of Appeal tended to engage in less EIP.

**Table 7:** Multiple regression of EBPAS-36 plus therapist and facility characteristics on EIP and CCR.

Significant Stepwise Predictor	*R*-Squared (Adjusted *R*^2^)	Final Standard β	t	*P*
Stepwise forward on EIP				
Job Security	.099 (.093)	.270	3.70	<.001
Openness	.153 (.142)	.281	3.63	<.001
Specialty certifications	.183 (.168)	.185	2.58	.011
Appeal	.204 (.183)	−.152	−1.99	.048
Backward elimination on EIP				
Openness	NA	.411	3.686	<.001
Job Security	NA	.249	3.786	<.001
Specialty Certifications	NA	.906	2.438	.016
Final model	.194 (.179)			<.0001
Stepwise forward on CCR				
Job Security	.161 (.156)	.364	4.99	<.001
Openness	.195 (.184)	.187	2.57	.011
Backward elimination on CCR				
Job Security	NA	.286	4.082	<.001
Feedback	NA	.181	2.055	.042
Professional Education	NA	−.165	−2.171	.031
Limitations	NA	.202	2.022	.045
Final model	.225 (.205)			<.001

Abbreviations: CCR, critical clinical reasoning; EBPAS-36 Evidence-Based Practice Attitude Scale-36; EIP, evidence-informed practice; NA, not applicable.

Predictors of CCR are shown at the bottom of table 7. Both stepwise forward and backward elimination regression found that Job Security was a significant predictor of CCR. Stepwise regression found that a single variable—Openness—further predicted CCR, while backward elimination supplanted Openness with three antecedent variables: Feedback, Professional Education (highest degree), and awareness of Limitations (of EBP), making a slightly strong predictive model (*R*^2^=.225 rather than .194). This pattern suggests that antecedent Professional Education and Feedback can affect Openness, and all appear to affect CCR.

## Discussion

*Aim 1.* Therapists’ habits of good, explicit, abductive reasoning—CCR—are by far the strongest predictors of engagement in self-regulated EIP. This finding replicates the results found in previously.^7^^,^^21^ The result is consistent with the classic definition of EBP as involving clinical expertise in application of best evidence clinical practice.^1^

*Aim 2.* As hypothesized, perceived Job Security was a significant predictor of both EIP and CCR. Openness (to new, research based, and manualized interventions) was related to CCR. This relationship is influenced by collinear attitudinal factors—Feedback and awareness of Limitations of evidence. Clinicians’ awareness of typical Limitations of evidence was related to *greater* engagement in EIP. Perceived Organizational Support was a significant predictor of both EIP and CCR, a result consistent with prior studies of EBP.

Specialty Certification proved to be the most significant educational predictor of EIP. A possible explanation is that obtaining Specialty Certifications reflects the commitment to lifelong learning of the therapist. Another is that Specialty Certifications tend to provide advanced training that targets specific, evidence-based, and clinically relevant factors that improve the management of actual client problems, emphasizing professional reasoning and decision-making activities required to apply and modify interventions to a specific client.

The reported lower frequency of engagement by therapists who have obtained terminal clinical degrees is consistent with the findings of Benfield and Jeffery (2022).^7^ These findings may be explained by the findings of others who suggest that the educational design of clinical doctorate curricula emphasize procedural knowledge and minimize explicitly development of skills and habits supporting lifelong learning and professional adaptability—specifically, developing the skills and habits of critical reflection, preparing for future learning and spirit of inquiry.^41^, ^42^, ^43^, ^44^, ^45^, ^46^

Several other factors theorized to affect evidence-related practices, however, did not predict recurrent self-regulated EIP activities, including level of education, profession, years of experience, facility size, and most subscales of the EBPAS-36. One reason for this might be the limited statistical power of the study: a larger sample or a different research design might detect significant effects of some of these variables.

There are other possible explanations too. Both dimensions of EIPT are different from EBP itself. Accredited clinical education programs explicitly aim at producing a defined level of “clinical competence” for all certified therapists. To the extent that they succeed in producing a uniform level of competence and skill, predictive correlations will be attenuated: correlations with a constant are zero. While years of clinical experience are no doubt required to develop clinical expertise, clinical experience may involve making clinical decisions primarily on tradition, what others do, pragmatism, and financial realities rather than reflection that enhances learning from clinical experience and outcomes or relevant research. Basing decisions primarily on traditional, pragmatic, or general “Appeal” tended to slightly impair EIP, at least after controlling for collinear factors (table 7). Moreover, clinical education programs may emphasize increasing knowledge; while knowledge per se is surely essential to achieving clinical expertise, studies have shown that increasing knowledge alone may well be insufficient to change clinical performances.^12^^,^^24^^,^^47^, ^48^, ^49^, ^50^ Finally, it is not uncommon for clinical education programs to emphasize clinical guidelines based on clinical tradition or authorities rather than controlled studies (eg, RCTs) and emphasize obedience to guidelines or fidelity of implementation rather than the full process of identifying evidence relevant to varied client problems and the complex, sequential abductive clinical reasoning processes of trying a treatment, altering approaches to fit the person and environment, checking responses, considering needed modifications. Knowledge taught in classrooms needs to be integrated with actual clinical evaluation, decision-making, and treatment processes; EIP and especially CCR emphasize routine aspects of this clinical process.

*Aim 3.* Preceding findings improve our understanding of processes that lead to greater engagement in EIP. Most important of these findings is the high correlation of engagement in CCR with engagement EIP activities. This suggests that EIP and EIPT implementation efforts in rehabilitation should actively involve and enhance therapists’ CCR. In other words, implementation efforts that increase therapists’ engagement in high quality CCR should, ceteris paribus, have a greater effect on self-regulated EIP/EBP than those that do not. This suggestion is consistent with previous reports that interventions to prepare clinicians for knowledge translation lead to greater sustained engagement in EBP 2 years after the initial educational seminar^51^ and adds to the growing literature on the need to change nonreflective routines among health care professionals.^24^^,^^52^

Clinicians’ awareness of the actual limitations of evidence is not necessarily a barrier to sustained EBP: such awareness is in fact associated positively with greater CCR and is not negatively associated with EIP engagement (table 4). To understand this result, it is essential to understand the result does not apply to global undiscriminating attitudes or beliefs that EBP is generally limited in its application to rehabilitation therapies. Rather the finding applies to awareness of comorbidities and specific, common, actual limitation of EBP, namely that RCTs focus somewhat narrowly on patients with common diagnoses, do not enroll clients with multiple problems or comorbidities, and are not individualized. Astute professional clinical reasoning is necessary to adapt and individualize treatment as part of the EBP process.^6^^,^^53^ Again, an implication is that education and knowledge translation efforts that focus entirely on obedience to manual protocols regardless of variations between clients, their problems or circumstances may be less likely to be successful at increasing sustained general EIP and CCR practices by therapists than efforts that that also address reasoning processes needed to individualize treatment.

Also, it is common to teach therapists to search for evidence when they encounter a clinical dilemma.^54^ While such a teaching strategy can make sense, results here suggest that teaching relevant EIPT habits—recurrently asking questions, searching, appraising, and integrating evidence from both relevant research and other therapists—may be more successful in enhancing therapists’ engagement in EBP. Knowledge should be applied when it is appraised as strong and relevant, not just when encountering uncomfortable clinical situations; this view is consistent with others’ call for developing the spirit of inquiry among health professionals generally.^55^, ^56^, ^57^

Past EBP implementation efforts have often employed administrative mandates or directive educational strategies, monitoring compliance fidelity using yes/no audit counts.^6^ Such efforts do not address reasons for modification of treatment protocols to fit individual clients. While audit and feedback may improve health professional performance to some degree, it works better if it focuses on important areas for improvement and includes tips for making changes and other supports.^17^ Documenting reasons for modifications of standard treatment protocols is critical to understanding variations in EBP application in implementation and quality of care studies.

### Future research and development

Continued research is needed on methods of improving best practices in rehabilitation therapies, including both studies focused on implementation of protocols with strong evidence base (specific EBPs) and studies of methods to enhance therapists’ habitual self-regulated EIP and their CCR habits. Specialty certification can involve testing knowledge and skills on evidence-based clinical practices for identified patient problems, rather than general principles or good ideas. Clinical education interventions such as grand rounds, which are not always part of education of rehabilitation therapists, involve sharing decision-making between practicing clinical educators and advanced students or residents. The “active ingredients” in advanced specialty certification warrant study, as such factors appear to be more effective at improving self-regulated EIP activities than other educational practices. Journal clubs for both students and practicing therapists involve sharing ideas from published research and clinical practice and should also promote EIP and CCR.

Educational interventions might also do well to draw from the literature on adult transformational learning.^52^ Habits of engaging in critical clinical reasoning may well be an active and essential ingredient in the transformative learning process needed to substantially improve the skillful application of best evidence by rehabilitative therapists. Future research on implementation strategies to increase the quality and effectiveness of rehabilitative care delivery should target therapists’ engagement in both CCR as well as EIP.

Additionally, future research on EBP implementation should address predictive factors identified here, such as specialty certification, Feedback, Openness, and Job Security, and problems associated with nonreflective clinical decision-making. Future research should examine the role of critical clinical reasoning in uptake, fidelity, and sustained use of EBPs. Educational interventions to improve practices should naturally focus on learning new skills, but they can also target learning activities which instill enduring engagement in EIP and CCR processes. Clinicians need explicit instruction and practice to employ the reflective professional reasoning schema that enhances the learning and application of new practices. Finally, therapists who already engage in high levels of EIP and CCR should be recruited to lead efforts to advance evidence-related quality and effectiveness of care.

### Study limitations

Because little previous work has been done on therapists’ self-regulated EIP and CCR and limited sample size, we aimed to identify only outstanding linear predictors of EIP or CCR, controlling for collinear confounders in the dataset. The sample size limited the number of valid statistical tests that can be performed. As the study has limited statistical power, false negatives are a risk. Prior relevant knowledge was insufficient to propose a definite a priori model, and surely there are other factors, not in our dataset, affecting high quality, effective clinical care. Replication in other samples—for example, in nonprofit facilities—would be desirable. RCTs are needed to clarify and confirm actual causal relationships.

EIPT focuses on therapists’ habitual activities, but there are also more specific domains of professional expertise and care quality involving technical skills, performances, knowledge, properly appraising strength and relevance of evidence, and fidelity of implementation of specific EBPs. Educational and policy intervention as well as research require focus on these more specific domains. Observational rather than self-report measures may be required.

## Conclusions

This study has identified educational factors (eg, specialty certification) and certain attitudes (eg, Openness and Job Security) related to therapists’ engagement in recurrent, self-regulated evidence application activities. Therapists’ engagement in reflective, abductive CCR is an essential component of the total process of their application of good evidence and learning from clinical experience. Future research to develop and test new implementation strategies should consider the multiple factors related to EIP and CCR in this study and a focus on the therapist own self-regulated activities to identify and apply best evidence and good clinical reasoning. Findings suggest that implementation strategies that successfully engage therapists’ abductive CCR are more likely also to increase their self-regulated EIPs than strategies that do not.

## Suppliers

a. REDCap, version 14.2; Vanderbilt University.
b. SPSS, version 24; IBM.

## Disclosure

The investigators have no financial or nonfinancial disclosures to make in relation to this project.
